# Editorials

**Published:** 1898-03

**Authors:** 


### HOMŒOPATHIC PHYSICIAN,

A MONTHLY JOURNAL OF

HOMŒOPATHIC MATERIA MEDICA AND CLINICAL MEDICINE.

"If our school ever gives up the strict inductive method of Hahnemann, we are lost, and deserve only to be mentioned as a caricature in the history of medicine."—CONSTANTINE HERING.

Vol. XVIII. MARCH, 1898. No. 3.

### EDITORIALS.

SILICEA.—This remedy has ulcers on the vermilion border of the lower lip. This is similar to Nitric-acid.

Natrum-muriaticum has fever blisters on the vermilion surface of the lips.

Several other remedies have these fever blisters.

The Editor once talking to Dr. Lippe on the subject of fever blisters upon the lips, years after he had ceased to lecture, gave the following list of remedies from Jahr:

Alum., Ammon-c., Ammon-mur., Arsen-a., Aur., Bell., Bry., Canth., Carbo-a., Carbo-veg., Caust., Cicuta, Clematis, Conium, Graphites, Hellebore, Hepar., Kali-carb., Lamium, Mag-carb., Mag-mur., Manganese, Mercurius, Muriatic-acid, Natrum-carb., Natrum-mur., Natrum-sulph., Nitrum, Paris, Phosphor., Platina, Ratanhia, Rhododendron, Rhus-tox., Sarsaparilla, Senega, Senna, Sepia, Silicea, Staphisagria, Sulphur, Valeriana, Veratrum, Zinc.

Silicea has caries of the lower jaw; this is very characteristic of Phosphorus. Caries of the lower jaw is reported to occur in the employees in match factories where phosphorus is used as an ingredient of the match-heads.

Silicea has toothache made worse by drawing cold air into the mouth, and Calcarea has toothache made worse by drinking cold water.

Silicea has sensation of a hair lying on the forepart of the tongue, and Kali-bichrom has sensation of a hair in the throat. Natrum-muriaticum has hair growing out of the tongue, and Arsenic has sense of hair in the throat like Kali-bichrom.

Under Silicea, when swallowing food, it easily gets into the posterior nares. This is a keynote of Dr. Guernsey.

Silicea has want of appetite and excessive thirst. Sulphur has the same symptom.

The Silicea patient has aversion to warm cooked food. He desires only cold things. Phosphorus has this same symptom. Lycopodium has aversion to boiled warm food.

Silicea has sour eructations after eating, and so has Sepia. Calcarea also has this symptom.

Silicea has vomiting after drinking. So also have Arsenicum, Ipecac, and Veratrum.

Silicea has vomiting as soon as he moves.

Silicea has sensitiveness of the pit of the stomach to pressure, and so has Nux-vomica. The Nux-vomica patient gets soreness of the epigastric region and upper part of the abdomen from coughing.

Silicea has hard, bloated abdomen, and Calcarea has swelling of the abdomen like a saucer turned bottom up. This is Dr. Guernsey's keynote.

Silicea has colic, with constipation. The pain is cutting. This is a characteristic.

Silicea has colic, with yellow hands and blue nails. This is a characteristic of Silicea.

Silicea has swelling and induration of the liver. This is similar to Murcurius and Lachesis.

Silicea has incarceration of flatulence difficult to discharge. If it be discharged it is very offensive. Lycopodium flatulence is not offensive.

Silicea has rumbling of flatulence in the abdomen. So also has Lycopodium. It is Guernsey's keynote for Lycopodium.

---

Vaccination in the Light of the Royal British Commission.—The Editor has received two or three letters from distinguished subscribers protesting against the further publication of the evidence upon the subject of vaccination. The tone of these communications indicates a certain misunderstanding on the part of the writers, and of objection to having the testimony placed within reach of those who are interested. In view of the fact that these objectors are homœopathic physicians, their opposition is somewhat surprising, and induces the Editor to take formal notice of their protests.

The fact that vaccination is doing mischief of a very grave character in the community is accumulating very rapidly. This evidence, with the tendency to extend the operation of the vaccination principle to the treatment of diphtheria and to many other diseases, has excited inquiry as to the scientific foundation of vaccination. These inquiries are very difficult to answer. The Editor considers himself fortunate in having obtained the desired answers in the very remarkable and able papers of Dr. Leverson, which are a condensation of the elaborate and exhaustive investigation ordered by that distinguished body, the British Parliament. The Editor thought the revelations in these papers were so astounding that he was conferring a favor on his readers by publishing them. In view of these protests, however, he desires to receive from the profession a full and free expression of views upon the subject, and if the writers so desire their papers will be published.

